# Effects of myopia on different areas and layers of the macula: a fourier-domain optical coherence tomography study of a chinese cohort

**DOI:** 10.1186/s12886-015-0080-5

**Published:** 2015-08-01

**Authors:** Zhennan Zhao, Xingtao Zhou, Chunhui Jiang, Xinghuai Sun

**Affiliations:** Department of Ophthalmology and Vision Sciences and Key Laboratory of Myopia of State Health Ministry, Eye and ENT Hospital, Shanghai Medical College, Fudan University, Shanghai, 200031 China; Department of Ophthalmology, No. 5 People’s Hospital of Shanghai, Shanghai, 200240 China

**Keywords:** Optical coherence tomography, Macular thickness, Inner and outer layer, Myopia

## Abstract

**Background:**

To explore the changes in thickness in different areas and layers of the macula under different refractive conditions.

**Methods:**

Consecutive participants were enrolled in the study. Optical coherence tomography (OCT) images were obtained using a spectral-domain system. We studied the relationships of the full, inner and outer retinal thicknesses of the fovea, parafovea and perifovea relative to the spherical equivalent (SE) and axial length (AL).

**Results:**

We included 107 eyes; the analysis revealed that the men had thicker foveas (man vs women: 236.70 ± 18.97 μm vs 247.25 ± 16.70 μm *P* = 0.002) and inner parafoveas (man vs women: 128.35 ± 8.80 μm vs 122.78 ± 6.75 μm *P* < 0.001) than the women had. Full foveal thickness was not correlated with AL or SE (all *P* > 0.05), whereas the full para- and perifoveal thicknesses had significant negative correlations with AL (r = –0.243 P = 0.006 and r = –0.446 *P* < 0.001, for para- and peri- respectively) and positive correlations with SE (r = 0.346 *P* < 0.001 and r = 0.464 *P* < 0.001, for para- and peri- respectively). Regarding the layers, the thickness of the inner layer of the fovea increased with the degree of myopia. The average inner and outer layer thicknesses of the para- and perifovea decreased with the degree of myopia (with the SE para, b = 0.307, peri b = 0.457; with the AL para, b = –0.266, peri b = –0.454),with the perifoveal thicknesses decreasing more rapidly.

**Conclusions:**

Macular thickness changes with the degree of myopia; however, the different areas and different layers change in different manners.

**Electronic supplementary material:**

The online version of this article (doi:10.1186/s12886-015-0080-5) contains supplementary material, which is available to authorized users.

## Background

Myopia has a high prevalence of approximately 80 % in Asia and approximately 25 % in other countries [1–3]. According to histological studies, the retina becomes atrophic and degenerates in myopic eyes, particularly at the posterior pole [4], and these changes are associated with a high frequency of macular abnormalities [5, 6]. As a result, monitoring the macular thickness in myopic eyes is of importance. Optical coherence tomography (OCT), which provides high-resolution retinal images, measures retinal thickness with high repeatability [7]. Findings on the relationship between refractive error/AL and macular thickness have been inconsistent among studies [8–14]. Few of these studies have focused on changes in thicknesses of the (inner/outer) retinal layers. Some conditions, such as open angle glaucoma (OAG), which has been found to have a much higher incidence in highly myopic eyes [15, 16], affect the inner layers of the retina more prominently [17, 18]. To explore the influence of myopia on the macula, including its different layers, a group of healthy young Chinese subjects with myopia was recruited. Macular thickness was evaluated by Fourier-domain OCT; the analysis focused on the thicknesses of different areas and layers of the macula and on potential correlations with the axial length (AL) and spherical equivalent (SE).

## Methods

The participants were consecutively enrolled from the Laser Center of EENT Hospital, Fudan University, in Shanghai, China, from July to December 2010. The participants all underwent a complete ophthalmologic examination, which included the following components: determination of best-corrected visual acuity; slit-lamp biomicroscopy; refraction measurement using autorefraction and refinement by an experienced optometrist; calculation of the SE using the spherical diopter (D) plus one-half of the cylindrical dioptric power for later analysis; a dilated fundus examination using a three-mirror contact lens; intraocular pressure (IOP) measurement using non-contact tonometer measurements; AL measurements using an IOLmaster instrument (Carl Zeiss Meditec, Inc., Jena, Germany); and a visual field test using automated static perimetry (Humphrey Field Analyzer II with a standard 30–2 program, Carl Zeiss Meditec, Dublin, CA, USA). The medical and family histories of the patients were collected.

The inclusion criteria were best-corrected visual acuity of 16/20 or better and myopia without other eye diseases. The exclusion criteria were as follows: a prior history of ocular surgery or trauma; diabetes mellitus; best-corrected visual acuity <16/20; IOP >21 mmHg; evidence of a reproducible visual field (VF) defect (with a significant standard deviation [SD] at the <5 % level or abnormal results in the glaucoma hemifield test) in either eye; unreliable VF test results (>15 % false positives or false negatives or >20 % fixation losses); a family history of glaucoma in a first-degree relative; signs of myopic degeneration or a pathological form of myopia; and other ophthalmic diseases, with the exception of refractive error and peripapillary atrophy. According to their refraction error, the participants were divided into the following four groups: low myopia (LM, <–3.0 D), moderate myopia (MM,–3.00 D to–6.00 D), high myopia (HM,–6.00 D to–10.00 D) and super high myopia (SHM, > −10.00 D). This research was approved by the Institutional Review Board of the Eye and Ear, Nose, and Throat (EENT) Hospital of Fudan University (Shanghai, China), and followed the tenets of the Declaration of Helsinki. Informed consent was provided by each subject.

A spectral-domain system, the RTVue OCT (RTVue-100 Optovue, Inc., software version: 2.0.5.39) system, was used for the study. With each participant, both eyes were examined at the same visit. The OCT images of the pupils, which were dilated, were obtained using the EMM5 scan pattern. Images with a signal strength index (SSI) greater than 40 were saved, and images with obvious misalignment of the interface detection algorithm or with apparent decentration were excluded. For each scan pattern, two series of images were saved, and the series with the higher SSI was used for further analysis. The RTVue automatic system provides images of the full, inner and outer layers of the macula for 9 map sectors, as defined by the Early Treatment Diabetic Retinopathy Study (ETDRS), and full retinal thickness was measured from the internal limiting membrane (ILM) to the middle of the retinal pigment epithelium (RPE). The retina between the ILM and the outer boundary of the inner nuclear layer (INL) was defined as the inner retina and the retina between the outer boundary of the INL and the middle of the RPE as the outer retina (Additional file 1: Figure S1). Foveal thickness referred to the average thickness of the retina at the 1-mm ring on OCT retinal thickness maps, the parafoveal (the ring at 1–3 mm) thickness was the average of the thicknesses of the four parafoveal sectors (superior, temporal, inferior and nasal), and the perifoveal (the ring at 3–5 mm) thickness was the average of the thickness of the four perifoveal sectors (superior, temporal, inferior and nasal) (Additional file 1: Figure S2).

The right eye of each patient was used for the data analysis. The statistical analysis was performed using SPSS software, version 17.0 for Windows. Student’s *t*-test was used to analyze the differences in macular thickness between male and female subjects. Pearson’s correlation coefficients were used to evaluate the relationships of the SE and AL with macular thickness. Multivariable linear regression analysis was used to investigate the tendency of macular thickness to change with the SE and AL. One-way analysis of variance (ANOVA) and the Scheffe post hoc multiple comparisons test were used to compare the differences among the four refraction groups. A *P* value less than 0.05 was considered statistically significant.

## Results

Based on the inclusion and exclusion criteria, a total of 107 eyes (107 subjects) were included in this study, with a mean age of 22.56 ± 5.15 years old (16–35 years), a mean SE of –6.73 ± 3.51 D (from –0.25 D –18.25 D), and an average AL of 26.25 ± 1.57 mm (22.31 –31.21 mm). A strong correlation was found between AL and SE (r = -0.893; *P* < 0.001).

According to the SE, the 107 eyes were divided into four groups, and detailed information on the four refractive groups is listed in Table 1. A total of 17 subjects were in the low myopia group (<–3.0 D), 31 were in the moderate myopia group (–3.00 D to –6.00 D), 43 were in the high myopia group (–6.00 D to -10.00 D), and 16 were in the super high myopia group (> −10.00 D). The four groups had similar ages (*P* = 0.267) and sex (*P* = 0.061) distributions, with different SE (*P* < 0.001) and AL (*P* < 0.001) distributions.

**Table 1:** Characteristics of the subjects from different myopic groups

Variable	Low Myopia Mean ± SD	Moderate Myopia Mean ± SD	High Myopia Mean ± SD	Super High Myopia Mean ± SD	
N	17	31	43	16	P-value
Age (year)	22.59 ± 5.17	22.90 ± 5.46	21.58 ± 4.23	24.50 ± 6.47	0.267
Gender	10 M, 7 F	20 M, 11 F	20 M, 23 F	7 M, 9 F	0.061
SE (D)	−1.63 ± 0.89	−4.59 ± 0.93	−8.24 ± 1.15	−12.22 ± 1.77	<0.001
AL (mm)	24.23 ± 0.99	25.62 ± 1.11	26.75 ± 0.88	28.17 ± 1.27	<0.001
IOP (mmHg)	14.11 ± 3.34	12.75 ± 3.16	14.06 ± 2.75	14.12 ± 2.58	0.219
MD (dB)	−3.16 ± 3.49	−1.56 ± 2.36	−1.61 ± 0.75	−0.86 ± 1.56	0.422
PSD (dB)	1.81 ± 3.7	1.82 ± 2.34	2.67 ± 0.58	1.64 ± 0.22	0.945

*IOP* intraocular pressure, *AL* axial length, *SE* spherical equivalent, *MD* mean deviation, *PSD* pattern standard deviation

The thickness measurements of the different macular parts and layers are listed in Table 2. The full para- and perifoveal thicknesses were significantly thinner in the high and super high myopic eyes than in the low and moderate myopic eyes (LM/MM > HM/SHM [all *P* < 0.001]) (Table 2); the full foveal thickness was not significantly thinner. The full foveal thickness was not correlated with AL or SE, whereas the full parafoveal and perifoveal thicknesses had negative correlations with AL (r = –0.243 *P* = 0.006 and r = −0.446 *P* < 0.001) and positive correlations with SE (r = 0.346 *P* < 0.001 and r = 0.464 *P* < 0.001) (Table 3). Multivariable linear regression analysis found that full thickness of the perifovea tended to decrease more with the degree of myopia than did that of the parafovea; i.e., farther from the fovea, the macular thickness decreased more with the progression of myopia (with the SE para, b = 0.307, peri b = 0.457; with the AL para, b = -0.266, peri b = −0.454) (Table 4, Additional file 1: Figure S3).

**Table 2:** Macular thickness in different myopic groups

Parameter	Low Myopia Mean ± SD	Moderate Myopia Mean ± SD	High Myopia Mean ± SD	Super High Myopia Mean ± SD		
n	17	31	43	16	P-value	Post hoc^a^
Macular Thickness (μm)						
Full Layer						
Fovea	239.42 ± 13.33	242.17 ± 18.77	243.98 ± 18.74	238.09 ± 22.63	0.665	-
Parafovea	316.52 ± 14.48	314.81 ± 11.66	307.81 ± 15.13	296.43 ± 19.60	<0.001	LM/MM > HM/SHM^*^
Perifovea	294.63 ± 11.60	284.10 ± 11.58	277.09 ± 14.13	266.26 ± 18.92	<0.001	LM/MM > HM/SHM^*^
Inner Layer						
Fovea	69.54 ± 8.11	71.64 ± 11.85	74.56 ± 10.04	71.75 ± 8.84	0.309	-
Parafovea	129.71 ± 7.07	127.93 ± 7.55	124.56 ± 8.04	119.10 ± 7.55	<0.001	LM/MM > HM/SHM^*^
Perifovea	116.34 ± 3.75	110.86 ± 4.94	107.94 ± 6.44	103.49 ± 6.29	<0.001	LM/MM > HM/SHM^*^
Outer Layer						
Fovea	170.12 ± 6.86	170.35 ± 8.46	169.46 ± 10.29	165.87 ± 16.82	0.560	-
Parafovea	186.82 ± 9.06	186.91 ± 7.90	183.28 ± 9.33	176.80 ± 15.82	0.009	LM/MM > HM/SHM^*^
Perifovea	178.53 ± 8.97	173.24 ± 7.80	169.17 ± 8.95	162.13 ± 15.63	<0.001	LM/MM > HM/SHM^*^

^a^Multiple comparisons among the four refraction groups

^*^ *P* < 0.05

*LM* low myopia; *MM* moderate myopia; *HM* high myopia; *SHM* super high myopia

**Table 3:** The relationships between macular thickness and age, axial length, and spherical equivalent

	Spherical Equivalents		Axial Length		Age	
	r	P-value	r	P-value	r	P-value
Macular Thickness						
Full Layer						
Fovea	−0.068	0.471	0.116	0.220	0.104	0.271
Parafovea	0.346	<0.001	−0.243	0.006	0.115	0.222
Perifovea	0.464	<0.001	−0.446	<0.001	0.091	0.335
Inner Layer						
Fovea	−0.177	0.060	0.234	0.012	0.038	0.688
Parafovea	0.353	<0.001	−0.220	0.019	0.058	0.540
Perifovea	0.527	<0.001	−0.519	<0.001	0.101	0.283
Outer Layer						
Fovea	0.057	0.552	−0.029	0.760	0.150	0.116
Parafovea	0.256	0.006	−0.198	0.036	0.131	0.170
Perifovea	0.372	<0.001	−0.349	<0.001	0.084	0.378

**Table 4:** The regression coefficients of macular thickness with gender, age and spherical equivalent (A)/axial length (B)

A						
	Gender		Age		Spherical Equivalents	
	b	P-value	b	P-value	b	P-value
Macular thickness						
Fovea						
Full layer	0.345	<0.001	0.188	0.064	−0.088	0.336
Inner layer	0.334	0.001	0.112	0.230	−0.203	0.082
Outer layer	0.300	0.002	0.234	0.150	−0.055	0.555
Parafovea						
Full layer	0.292	0.001	0.202	0.063	0.307	0.001
Inner layer	0.333	<0.001	0.157	0.076	0.287	0.001
Outer layer	0.195	0.042	0.194	0.071	0.255	0.006
Perifovea						
Full layer	0.039	0.658	0.122	0.165	0.457	<0.001
Inner layer	−0.009	0.918	0.126	0.139	0.516	<0.001
Outer layer	0.072	0.443	0.123	0.184	0.372	<0.001
B						
	Gender		Age		Axial Length	
	b	*P*-value	b	*P*-value	b	*P*-value
Macular thickness						
Fovea						
Full layer	0.327	0.001	0.191	0.140	0.096	0.285
Inner layer	0.291	0.002	0.120	0.195	0.215	0.017
Outer layer	0.311	0.001	0.231	0.160	0.054	0.551
Parafovea						
Full layer	0.365	<0.001	0.200	0.062	−0.266	0.002
Inner layer	0.396	<0.001	0.152	0.089	−0.246	0.005
Outer layer	0.258	0.007	0.189	0.082	−0.219	0.018
Perifovea						
Full layer	0.144	0.102	0.113	0.195	−0.454	<0.001
Inner layer	0.104	0.216	0.111	0.185	−0.524	<0.001
Outer layer	0.159	0.089	0.117	0.207	−0.362	<0.001

**Fig. 1:**
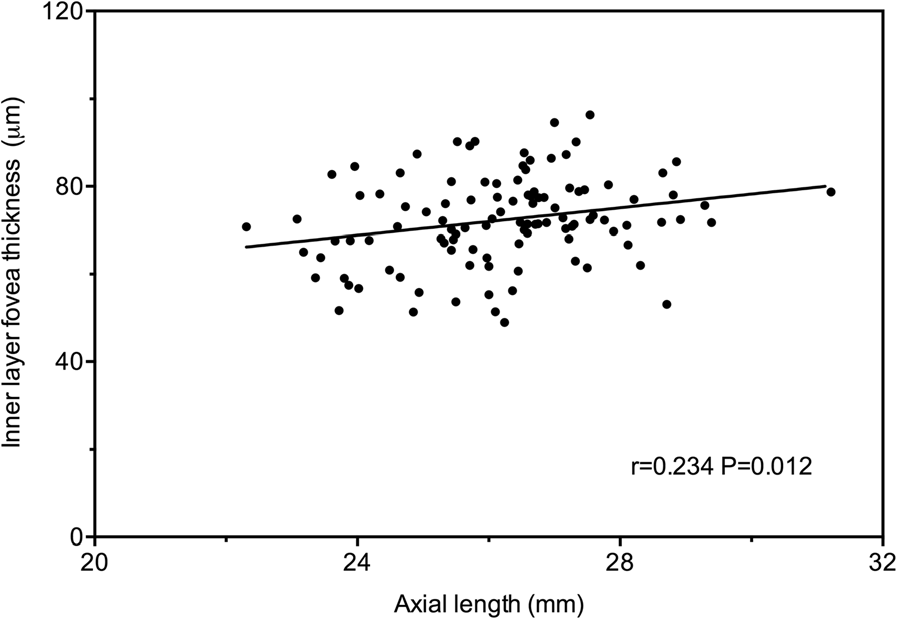
Scatterplots of inner layer foveal thickness against axial length (AL). The inner layer foveal thickness was significantly positively associated with the AL (r = 0.234, *P* = 0.012)

Regarding the different macular layers, the inner and outer thicknesses of the para- and perifoveal portions, and not of the foveal portion, were significantly thinner in the high and super high myopic eyes than in the low and moderate myopic eyes (LM/MM > HM/SHM [all *P* < 0.05]) (Table 2). Additionally, the analysis found that the thicknesses of the inner and the outer layer of the para- and perifovea showed negative correlations with AL and positive correlations with SE (Table 3), whereas the inner layer of the fovea showed a positive correlation with AL (r = 0.234, *P* = 0.012, Fig. 1). The inner and outer layers of the retina appeared to change in much the same manner as the para- and perifoveal areas. Compared with the parafoveal area, the inner and outer layers of the perifoveal area decreased more rapidly with the progression of myopia (Table 4, Additional file 1: Figure S4).

Additionally, multivariable linear regression analysis found that the full, inner and outer layer thickness from the foveal as well as parafoveal part was correlated with gender, even after the correction of age and AL/SE (Table 4). Further study showed that the full, inner and outer thicknesses of the fovea, as well as the full and inner thicknesses of the parafovea, were higher in men than in women (all *P* < 0.05) (Table 5); however, no correlations were found between age and any of the macular thickness measurements (all *P* > 0.05) (Tables 3, 4).

**Table 5:** Background and macular thicknesses of male and female subjects

Characteristics n	Female Mean ± SD 50	Male Mean ± SD 57	*P*-value
Age, year	23.91 ± 5.73	21.29 ± 4.07	0.006
Spherical equivalent (D)	−7.09 ± 3.88	−6.38 ± 3.03	0.058
Axial length (mm)	26.13 ± 1.65	26.38 ± 1.52	0.404
Macular retinal thickness (μm)			
Full thickness			
Fovea	236.70 ± 18.97	247.25 ± 16.70	0.002
Parafovea	304.91 ± 13.10	314.26 ± 17.51	0.002
Perifovea	278.95 ± 14.56	281.46 ± 17.50	0.405
Inner layer thickness			
Fovea	69.74 ± 10.35	75.40 ± 9.37	0.03
Parafovea	122.78 ± 6.75	128.35 ± 8.80	<0.001
Perifovea	109.18 ± 6.18	109.63 ± 7.29	0.719
Outer layer thickness			
Fovea	166.82 ± 10.58	171.88 ± 9.71	0.010
Parafovea	182.14 ± 9.17	185.96 ± 11.27	0.052
Perifovea	169.91 ± 10.38	171.86 ± 11.08	0.340

## Discussion

In a cohort of young Chinese subjects, macular thickness was examined using Fourier-domain OCT. Further analysis found that different macular parts, as well as different macular layers, changed in different manners. Whereas foveal thickness was not correlated with AL or SE, the para- and perifoveal thicknesses were negatively correlated with AL and positively correlated with SE. Regarding the different layers of the retina, the inner layer in the fovea was positively correlated with AL, whereas the inner and outer layer thicknesses in the para- and perifoveal parts were negatively correlated with AL (positively with SE).

Macular thickness in high myopia has been studied; however, most of these studies have included subjects with relatively wide age ranges [8, 14] and have used time-domain OCT systems with limited resolution and speed [8–13]; few of these studies have examined the changes in thicknesses of the inner and outer layers separately. In this study, a Fourier-domain OCT system, with high speed and resolution, was used, and the macular area was covered by densely compacted scan lines with auto-segmentation so that the inner and outer layers of the retina could be measured separately. Because age has been reported to affect macular thickness [19], a cohort of young Chinese subjects of similar ages was recruited.

Full foveal thickness did not decrease in myopic eyes; however, the full para- and perifoveal thicknesses were negatively correlated with AL and positively associated with SE, and the farther from the fovea, the more the macular thickness decreased with the progression of myopia. These findings were consistent with the results of Wakitani [8], Lim [10] and Liu [20], suggesting that retinal thinning in myopia occurred more frequently in the peripheral part. Lam et al. [13] recently found that, at the outer ring (3–6 mm) and not at the inner ring (1–3 mm), macular thickness decreased in myopia. Also it was reported that while the photoreceptors and RPE aggregate thickness had a negative correlation with AL and SE at other area of the fundus, this kind of changing was not found at the fovea [21]. It is speculated that the peripheral retina is less resistant to traction and stretch, mainly because of the absence of large blood vessels [22]. At the posterior pole, it might be slightly different; the perifoveal retina, which is closer to the vascular arcade**,** decreased more rapidly with myopia than did the parafoveal part, which is far from the vascular arch. However, the parafoveal retina is thicker than the perifovea; therefore, lower resistance might still explain the finding.

Increased foveal thickness in myopic eyes has been reported in other studies [10–14, 23]. Tangential traction by the ILM or posterior vitreous cortex was hypothesized to be one of the reasons, whereas Luo suggested that increased foveal thickness might be caused by the high permeability of the RPE at the fovea [12, 24]. Our study found that increasing retinal thickness occurred predominantly in the inner layer, which appeared to support the theory of traction acting on the inner surface. Our subjects were relatively young, and reasons other than vitreoretinal traction by incomplete or abnormal vitreous detachment and liquefaction might be anticipated as well.

Whereas the thickness of the foveal inner layer increased with the degree of myopia, the inner and outer layer thicknesses of the para- and perifovea significantly decreased in myopic eyes. Wolsley [25] and Cheng [26] reported that, in myopic eyes, the mid-inner macular layer was thinner; however, the outer layer was not thinner [25, 26], and the inner and outer layers were thinner at more peripheral locations [26]. In those studies, only time-domain OCT systems were available, and one scan line was analyzed. In this study, a Fourier-domain OCT covered the entire macular area with a series of scans, and changes in retinal thickness in myopia might therefore have been more clearly revealed. We found that the inner and outer retina changed with similar tendencies in the para- and perifoveal area, with the perifoveal inner and outer layers decreasing more rapidly than the parafovea. This result is in agreement with our findings that the full perifoveal thicknesses were more negatively correlated with AL (positively with SE) compared with the parafovea (Table 4, Additional file 1: Figure S3 and Additional file 1: Figure S4).

Our results showed that women tended to have thinner foveas and inner parafoveas. Similar findings have been reported [10, 12, 13], consistent with the clinical findings that women have a higher risk of idiopathic macular holes [27], which have been suggested to begin with foveal thinning [28]. Some researchers have hypothesized that hormonal changes during menopause, from hysterectomy or from hormone replacement therapy might explain these findings in women [29], whereas other studies have suggested that the thicker fovea in men might be an early subclinical sign of vitreoretinal traction in highly myopic eyes [10]. In this study, we found foveal thinning in a young population, and neither menopause nor traction from partial vitreous detachment was very likely. Moreover, not only the inner layer but also the outer layer of the fovea was thicker in men. Consequently, the reasons for this finding in women should be studied further.

Detecting such changes in macular thickness by Fourier-domain OCT is helpful in understanding clinical findings in myopic eyes, and further studies for the mechanism underneath might help us to find ways to treat or even prevent the complications in highly myopic eyes. Also our results together with others [8–14, 21, 23–26] implied that caution must be taken when interpreting data of retinal thickness of different area as well as different layers of the macula measured by OCT in highly myopic eyes.

This study used a single-race cohort with a limited age range of subjects from a single hospital, and these factors might have resulted in bias; a multi-center study, including more elderly subjects, would improve our knowledge of the changes in different macular layers, as well as different parts of the macula, in myopic eyes.

## Conclusions

Our study found that macular thickness changes with the degree of myopia; however, the changes differ according to the macular regions and layers. Using macular thickness in the diagnosis and assessment of eye diseases requires attention to the refractive state and, in some situations, to the sex of the subject.

## Additional file

Additional file 1: Figure S1. The full, inner and outer layer of macular thickness. The RTVue OCT system automatically provides the full, inner and outer layer of macular thickness. From left to right, three white arrows indicated the middle of the retinal pigment epithelium (RPE), inner limiting membrane (ILM) and the outer boundary of the inner nuclear layer (INL), respectively. The full retinal thickness was measured from the ILM to RPE; the retina between the ILM and INL is defined as inner retina, the retina between INL and RPE is the outer retina. **Figure S2**. The Early Treatment Diabetic Retinopathy Study (ETDRS) map. Example of a macular thickness measurement by RTV-ue OCT in a subject’s right eye. The Average foveal thickness refers to the average thickness of retina at 1-mm ring on the OCT retinal thickness map; average parafoveal (ring at 1–3-mm) thickness is the average of thickness of all four parafoveal sectors (superior, temporal, inferior and nasal), average perifoveal (ring at 3-5 mm) thickness is the average of thickness of all four perifoveal sectors (superior, temporal, inferior and nasal). **Figure S3**. Graph showing the average full macular thickness vary with SE (A) and AL (B). The full thickness of the perifovea tended to decrease more with the degree of myopia than did that of the parafovea (with the SE para, b = 1.571, peri b = 2.105; Figure S3A; with the AL para, b = −2.446, peri b = −4.520; Figure S3B). **Figure S4**. Graph showing the average inner and outer layer macular thickness vary with SE (A) and AL (B). The inner and outer layer of the retina seems to change in much the same manner at the parafoveal (with SE inner b = 0.916, outer b = 0.873; Figure S4A; with AL inner b = −1.028, outer b = −1.309; Figure S4B) and perifovea area (with SE inner b = 1.120, outer b = 1.348; Figure S4A; with AL inner b = −2.136, outer b = −2.573; Figure S4B), but the inner and outer layers at the perifoveal area decreased more quickly with the progression of myopia. (DOC 22 kb)

## Abbreviations

OCT: Optical coherence tomography
SE: Spherical equivalent
AL: Axial length
OAG: Open angle glaucoma
IOP: Intraocular pressure
VF: Visual field
LM: Low myopia
MM: Moderate myopia
HM: High myopia
SHM: super high myopia
SSI: Signal strength index
ETDRS: Early treatment diabetic retinopathy study
ILM: Internal limiting membrane
RPE: Retinal pigment epithelium
INL: Inner nuclear layer
ANOVA: One-way analysis of variance
